# Modeling effects of diabetes and obesity co-morbidities in endometrial cancer development and progression

**DOI:** 10.1186/1753-6561-6-S3-P56

**Published:** 2012-06-01

**Authors:** Doris Benbrook, Erin Bishop, Elizabeth Nugent, Stan Lightfoot, Thavathiru Elangovan, Andy Long, Daniel Zhao

**Affiliations:** 1 Department of Obstetrics and Gynecology, Section of Gynecologic Oncology, University of Oklahoma Health Sciences Center, Oklahoma City, OK, 73104, USA; 2 Department of Pathology, University of Oklahoma Health Sciences Center, Oklahoma City, OK, 73104, USA; 3 Department of Biochemistry and Molecular Biology, University of Oklahoma Health Sciences Center, Oklahoma City, OK, 73104, USA; 4 Department of Statistics and Epidemiology, University of Oklahoma Health Sciences Center, Oklahoma City, OK, 73104, USA

## Background

Diabetes and obesity are well established risk factors for the development of endometrial cancer. We hypothesized that circulating cytokines associated with diabetes and obesity exert direct effects on primary human endometrial cell proliferation and transformation and worsen pathology in endometrial cancer patients.

## Methods

The effects of insulin and insulin like growth factors 1 and 2 (IGF1 and 2) on proliferation were measured in primary cultures of human endometrial cells using a cytotoxicity assay, while transformation was measured by soft agar colony formation and histologic evaluation of and organotypic model of endometrial carcinogenesis and chemoprevetion [1]. Akt phosphorylation status was measured by Western blot. Levels of leptin, adiponectin adipsin, c-peptide, tumor necrosis factor α (TNFα) chemokine (C-C motif) ligand 2 (CCL2, also called monocyte chemotactic protein-1 or MCP-1), Interleukin (IL)-1β, IL10, vascular endothelial growth factor (VEGF) and insulin in serum from 84 endometrial cancer patients were measured using multiplex magnetic bead assays (BioRad and Millipore). Glucose was measured using a kit (Cayman), and HOMA Scores were calculated. These biomarkers were compared with tumor stage, grade, recurrence of disease and age at diagnosis using Spearman Correlation.

## Results

Insulin, IGF1 and IGF2 caused dose-responsive induction of endometrial cell proliferation, which surprisingly was associated with loss of Akt phosphorylation on Ser473. Insulin treatment as a single agent induced anchorage-independent colony formation in the soft agar assay and histologic features of transformed cells in organotypic cultures. Insulin also enhanced these transformation measures induced by 7,12-Dimethylbenz(a)anthracene (DMBA) carcinogen. Elevated CCL2 in serum of endometrial cancer patients was significantly associated with worse stage of disease (r=0.262, p=0.018). Reduced serum TNFα was significantly associated with increased age at diagnosis (r=0.332, p=0.048). No other associations between biomarkers and patient characteristics were noted.

## Conclusions/discussion

Insulin, IGF1 and IGF2 have direct effects on normal endometrial cell proliferation and transformation potential implicating these cytokines in the increased risk of endometrial cancer associated with diabetes and obesity. The decreased TNFα found to be associated with increased age at diagnosis may be due to the known inverse association of TNFα with age and insulin levels. The association of elevated CCL2 with tumor stage suggests that direct effects of CCL2 on tumor and surrounding stromal cells over-rides CCL2 recruitment of T-lymphocytes into tumors to enhance tumor-specific immunity. Follow-up studies are planned to validate the biomarker results in an independent set of specimens and to evaluate the role of Akt signaling in insulin and CCL2 effects on endometrial carcinogenesis and progression.
